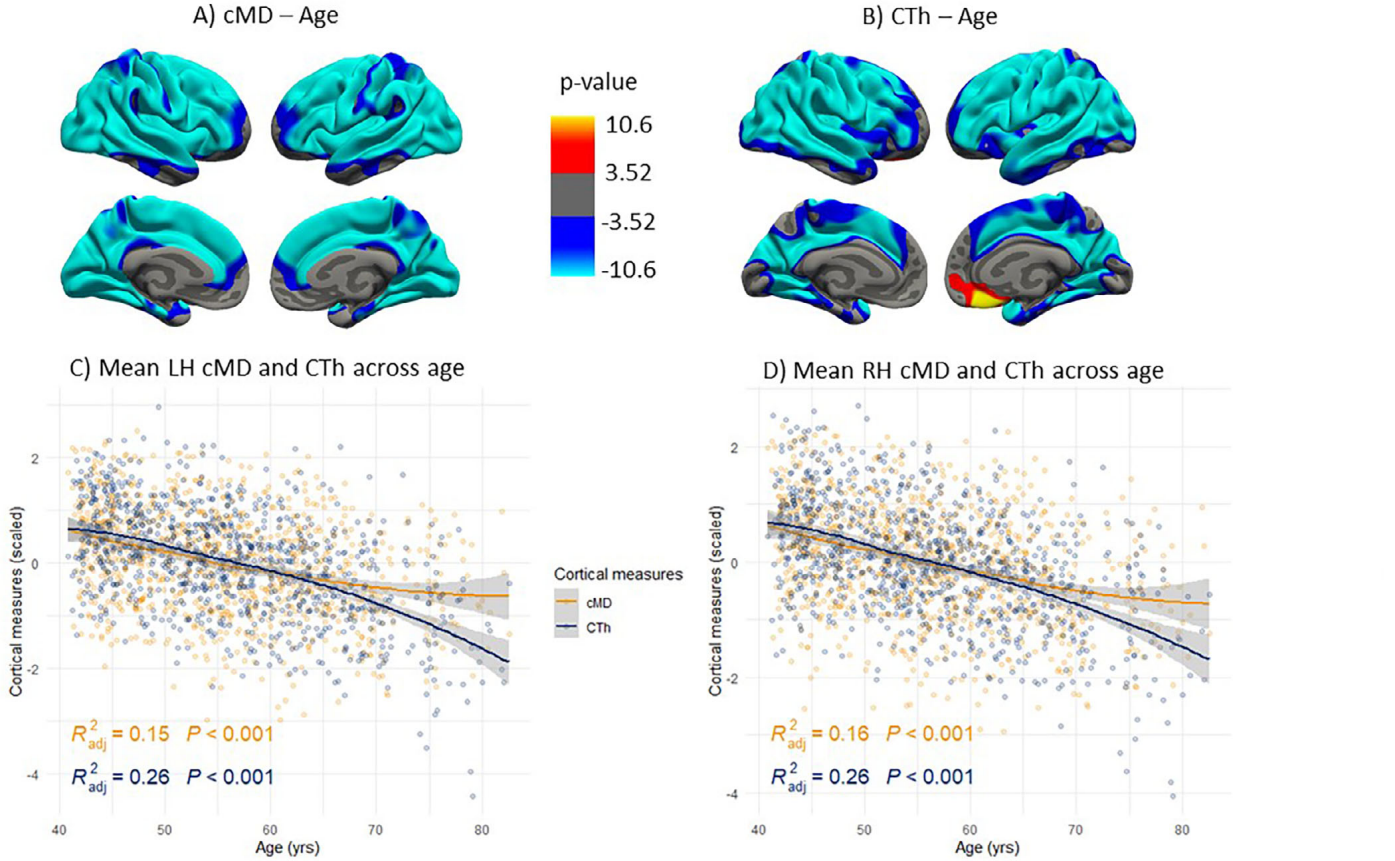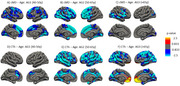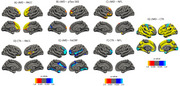# Cortical integrity changes with cognition, plasma biomarkers and *APOE4* status across the adult healthy lifespan

**DOI:** 10.1002/alz70862_109941

**Published:** 2025-12-23

**Authors:** Oriol Perera‐Cruz, Lídia Mulet‐Pons, Victor Montal, Cristina Solé‐Padullés, María Cabello‐Toscano, Ruben Perellón‐Alfonso, Gabriele Cattaneo, Javier Solana Sánchez, Vanessa Alviarez‐Schulze, Núria Bargallo, Juan Fortea, Josep Mª Tormos‐Muñoz, Alvaro Pascual‐Leone, Lídia Vaqué‐Alcázar, David Bartrés‐Faz

**Affiliations:** ^1^ Department of Medicine, Faculty of Medicine and Health Sciences and Institute of Neurosciences, University of Barcelona, Barcelona, Barcelona Spain; ^2^ August Pi I Sunyer Institute of Biomedical Research (IDIBAPS), Barcelona, Barcelona Spain; ^3^ Sant Pau Memory Unit, Department of Neurology, Institut d’Investigacions Biomèdiques Sant Pau ‐ Hospital de Sant Pau, Barcelona, Barcelona Spain; ^4^ Institut Guttmann, Institut Universitari de Neurorehabilitació adscrit a la Universitat Autònoma de Barcelona, Badalona, Barcelona Spain; ^5^ Fundació Institut d’Investigació en Ciències de la Salut Germans Trias i Pujol, Badalona, Barcelona Spain; ^6^ Unit for Cognitive Neuroscience, Institute of Neurosciences, University of Barcelona, Barcelona, Barcelona Spain; ^7^ Centre for Biomedical Research on Mental Health (CIBERSAM), Instituto de Salud Carlos III, Madrid, Madrid Spain; ^8^ Imaging Diagnostic Center Radiology Department, Hospital Clínic i Provincial de Barcelona, Barcelona, Barcelona Spain; ^9^ Centro de Investigación Translacional San Alberto Magno, Facultad Ciencias de la Salud, Universidad Católica de Valencia, Valencia Spain; ^10^ Hinda and Arthur Marcus Institute for Aging Research and Deanne and Sidney Wolk Center for Memory Health, Hebrew SeniorLife, Department of Neurology, Harvard Medical School, Boston, MA USA; ^11^ Department of Neurology, Harvard Medical School, Boston, MA USA

## Abstract

**Background:**

The relationship between cognitive age‐related changes and cortical macrostructural properties (i.e., cortical thickness [CTh]) has been extensively studied. However, less is known about the relation with microstructural characteristics (i.e., cortical mean diffusivity [cMD]) even though these are sensitive to preclinical phases of Alzheimer’s disease (AD). Furthermore, the relationship between macro‐ and microstructural measures with cognition, neurodegeneration and inflammation plasma biomarkers among healthy individuals has not been reported.

**Method:**

A total of 964 adults (age: 40‐82 years; 52% females) with normal neuropsychological profile and available structural and diffusion MRI data were included. FreeSurfer was used to obtain CTh maps. For cMD processing we used a homemade surface‐based approach after preprocessing the diffusion images with FSL. Surface maps were smoothed (FWHM=15) and the group vertex‐wise statistical maps were considered significant at *p* <0.05. The Preclinical Alzheimer’s Cognitive Composite (PACC) was used as the cognition measure. Plasma concentrations of phosphorylated tau (*p*‐Tau181), neurofilament light (NFL) and C‐reactive protein (hsCRP) were analyzed, together with *APOE4* status.

**Result:**

We identified a significant negative association between both cortical measures and age (Figure 1). cMD associations were more extensive at earlier ages (under 50 years), while CTh associations were greater at older ages (above 50 years) (Figure 2). cMD was positively correlated with PACC scores and both pTau‐181 and NFL concentrations in prefrontal regions, while the association was negative and more widespread for hsCRP (Figure 3). CTh results revealed smaller positive and negative clusters only for PACC and NFL, respectively (Figure 3). Correlating cMD with CTh differentiated somatosensory and associative areas with negative and positive correlations, respectively (Figure 3). This pattern was conserved after covarying for sex, age and biomarker data, and across age and risk (*APOE4* carrier) subsamples.

**Conclusion:**

We show that cMD, which might reflect neuronal density loss accompanying or even preceding atrophy, is able to capture microstructural cortical changes occurring during natural aging before CTh alterations. Indeed, it seems more sensitive to age‐related cognitive decline. Furthermore, our results suggest a pattern relating the two metrics conserved across different aging trajectories and opposite trends of the two cortical measures in relation to neurodegenerative biomarkers levels.